# A Comprehensive Review of Hollow-Fiber Membrane Fabrication Methods across Biomedical, Biotechnological, and Environmental Domains

**DOI:** 10.3390/molecules29112637

**Published:** 2024-06-03

**Authors:** Cezary Wojciechowski, Monika Wasyłeczko, Dorota Lewińska, Andrzej Chwojnowski

**Affiliations:** Nalecz Institute of Biocybernetic and Biomedical Engineering, Polish Academy of Sciences, Trojdena 4 Str., 02-109 Warsaw, Poland; mwasyleczko@ibib.waw.pl (M.W.); dlewinska@ibib.waw.pl (D.L.); achwoj@ibib.waw.pl (A.C.)

**Keywords:** hollow-fiber membranes, spinning parameters, membrane separation, biopolymers, retention, degradation, hydrolysis, fouling

## Abstract

This work presents methods of obtaining polymeric hollow-fiber membranes produced via the dry–wet phase inversion method that were published in renowned specialized membrane publications in the years 2010–2020. Obtaining hollow-fiber membranes, unlike flat membranes, requires the use of a special installation for their production, the most important component of which is the hollow fiber forming spinneret. This method is most often used in obtaining membranes made of polysulfone, polyethersulfone, polyurethane, cellulose acetate, and its derivatives. Many factors affect the properties of the membranes obtained. By changing the parameters of the spinning process, we change the thickness of the membranes’ walls and the diameter of the hollow fibers, which causes changes in the membranes’ structure and, as a consequence, changes in their transport/separation parameters. The type of bore fluid affects the porosity of the inner epidermal layer or causes its atrophy. Porogenic compounds such as polyvinylpyrrolidones and polyethylene glycols and other substances that additionally increase the membrane porosity are often added to the polymer solution. Another example is a blend of two- or multi-component membranes and dual-layer membranes that are obtained using a three-nozzle spinneret. In dual-layer membranes, one layer is the membrane scaffolding, and the other is the separation layer. Also, the temperature during the process, the humidity, and the composition of the solution in the coagulating bath have impact on the parameters of the membranes obtained.

## 1. Introduction

**Figure 1:**
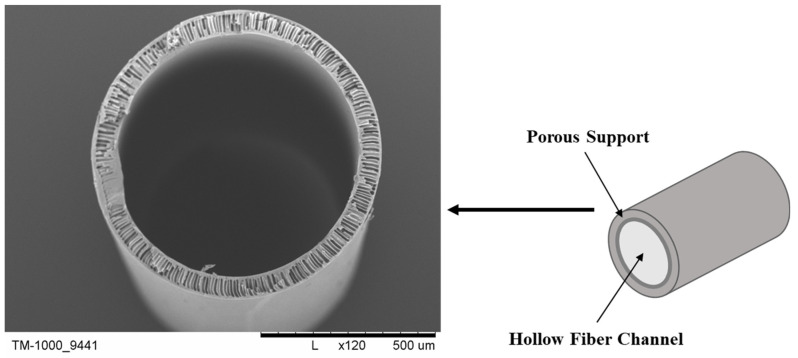
Schematic illustration (**right**) and SEM image of a hollow-fiber membrane (**left**). Scale bar: 500 µm.

Polymer hollow-fiber membranes, among all membranes, play an important role in their application in many industries. Hollow-fiber membranes (Figure 1) are thin structures with a porous construction. They can separate various substances based on their size and chemical properties, making them ideal for filtration applications. Their most important application is broadly understood to be environmental protection, i.e., treatment of municipal and industrial wastewater. In wastewater treatment, we most often initially use polypropylene (PP) membranes and then polysulfone (PSf) membranes. In seawater desalination, cellulose 2,5-acetate membranes applied on ceramic membranes are used. Sea water treatment and desalination is widely used in the water-poor rich Arab countries as well as in the countries of the Mediterranean basin. Membranes are used in the recovery of valuable compounds from industrial wastewater, as well as in the pharmaceutical industry for the separation and purification of medical drugs. Moreover, hollow-fiber membranes are useful in biotechnology, medicine, and other applications for their widespread use in blood purification, removing toxins through processes like dialysis. In blood purification and dialysis, membranes made of cellulose acetate (CA), regenerated cellulose (cuprophan), and PSf are used. In medicine, the most commonly used membranes are PP, PSf, and polyethersulfone (PES). Additionally, they hold promise as materials for bioartificial liver constructs, pancreatic islet transplantation, or cultivating various cell types [1,2,3,4,5,6,7,8,9,10,11,12,13]. Before application, membranes are placed into appropriate modules, which are then utilized in dialyzers or bioreactors [3,14].

The manufacturing process for hollow-fiber membranes is referred to as “spinning capillary membranes”, a commonly employed technique for producing micro- and ultrafiltration membranes featuring microscopic or nanoscale pores. Membranes require a special installation for their manufacture, the most important component of which is the hollow fiber-forming spinneret. This method most often produces membranes made of PSf, PES, polyurethane (PUR), polyvinylidene fluoride (PVDF), cellulose acetate (CA), and their derivatives or blends [3,4,5,12,15,16].

**Figure 2:**
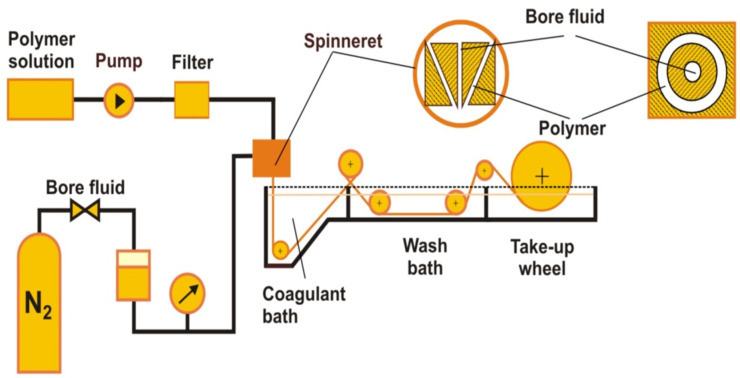
Schematic diagram of the spinning process for hollow-fiber membranes.

The membranes are obtained via a dry–wet spinning phase-inversion technique through extrusion of the polymer solution. The polymeric dope solution is extruded by syringe pumps through the spinneret. The bore fluid (mostly pure water) is supplied towards the spinneret via ambient gas pressure. The nascent hollow-fiber membranes are passed through an air gap (the distance between the spinneret and the coagulation bath; distance varying from 0 to 20 cm), which is drawn into coagulation baths and then transported over the rollers (Figure 2). The coagulant is pure water in the first bath and tap water in two other ones. A take-up wheel is used to collect the hollow fibers. The membrane spinning process is carried out at room temperature [12,15,17].

This review article presents various methods of obtaining polymeric hollow-fiber membranes produced using the dry–wet phase inversion method, outside the TIPS method, published in renowned specialized membrane publications in the years 2010–2020, such as the Journal of Membrane Science, Desalination, Polymer, Desalination of Water Treatment, and others. We tried to include all relevant methods of obtaining polymer membranes in the form of the shortest possible messages containing the method of modification and its result, referring interested persons to the articles included in the Reference section, where the given modifications are extensively presented.

## 2. Hollow-Fiber Membrane Preparation

### 2.1. The Spinning Process Parameters for Hollow-Fiber Membranes

#### 2.1.1. Changes in Polymer Extrusion Velocity, Pressure, Temperature, Bore Fluid, and Air Gap

In the course of hollow-fiber membrane production, a multitude of modifications can be applied. The first of these is the changing of the parameters in the process of obtaining membranes, such as the speed of extrusion of the polymer solution from the spinneret, the pressure at which the bore fluid is fed, the distance of the air gap, and the speed of winding the hollow fibers on the take-up wheel. Moreover, the process temperature, humidity, and solution composition in the coagulating bath have an impact on the parameters of the obtained membranes. By changing the parameters of the spinning process, we change the wall thickness and diameter of the membranes, which causes changes in their structure and, as a consequence, changes their transport/separation parameters [18,19,20,21,22,23,24,25,26,27,28].

Publications discussing the impact of changing parameters in the process of obtaining membranes, such as the speed of extrusion of the polymer solution from the spinneret, the pressure at which the bore fluid is fed, the distance of the air gap, and the speed of winding the hollow fibers on the take-up wheel, are listed below.

The first publication presents the production of polyaniline (PANI) capillary membranes for gas separation. The authors themselves synthesized the above-mentioned high-molecular-weight polymer. The membranes were obtained with the use of an in-house capillary spinning installation, where the influence of the air gap distance on the emerging membrane morphology was investigated. Tetrahydrofuran (THF) was used as the polymer solvent, which resulted in the formation of a loose skin layer. The optimal distance of the air gap was experimentally selected to obtain the maximum selectivity for O_2_/N_2_, H_2_/N_2_, and H_2_/CO_2_ [18].

In another publication, PUR capillary membranes obtained via melt spinning were exposed to hot air, which influenced their morphology and functional properties. The maximum average pore size was obtained at a draw ratio of 2 at 70 °C for 30 min. However, when the membrane was processed under stress-free conditions, the pore sizes on the outer surface of the membrane decreased, and the pure water flux decreased with increasing heat treatment temperatures. However, at a temperature of 150 °C, a dense and smooth external surface with a thick epidermal layer was obtained [19].

In the subsequent article, membranes were fabricated through the blending of PVDF with an amphiphilic Pluronic block copolymer. In this case, the addition of Pluronic/LiCl with a very low concentration of Pluronic was used. A small addition of 0.2% by weight Pluronic was enough to significantly change the morphology, efficiency, and mechanical properties of the obtained capillaries. In order to obtain capillaries with a high filtration efficiency, they are spun at a higher coagulant temperatures and at a high capillary take-off rate of 3% weight LiCl and 0.2% weight Pluronic [20].

Another article described the development of a new coaxial spinneret for producing capillary PES membranes via phase separation. The resulting membranes had two dense skin layers on both the outer and the inner surfaces. The structure of the membranes was easily controlled by regulating the flow of fluids to the membrane spinneret [21].

Another way to change the parameters and morphology of polyacrylic acid (PAA) membranes is to change the parameters of their spinning. The concentration of PAA, the composition of the core fluid, the flow rate of the solutions, the speed of membrane collection, the air gap distance, and the temperature of the coagulation bath were controlled. Based on the above changes, the mechanism of membrane morphology was explained [22].

A method of obtaining spiral capillary PES membranes was developed using the phenomenon of winding a liquid rope. In an experimental way, the parameters of membrane spinning were selected in such a way as to reflect the conditions in which spiral membranes are formed. It was observed that the increase in the air gap distance and the increase in the viscosity of the dope solution led to the formation of spiral membranes. On the other hand, strong solvents created an inner skin, which made the capillaries straight due to their quick gelation before curling [23].

Capillary membranes made of polyacrylonitrile (PAN) with the addition of alkaline lignin were made using the wet spinning technique. The membranes contained mainly mesopores and macropores. The influence of stretching the membranes during spinning on the structure and mechanical strength of the capillaries was investigated [24].

In the preparation of capillary membranes from polystyrene-block-poly(4-vinylpyridine) (PS-b-P4VP), the influence of spinning parameters on the morphology of the membranes formed was investigated. The following parameters were tested and changed: solution concentration and viscosity, extrusion pressure in the spinneret, and air gap distance [25].

PUR capillary membranes were produced and treated with heat under various conditions. It was observed that the pore size decreased with increasing treatment temperatures, while stretching increased the pore size. The best water flow through the membranes was obtained at the treatment temperature of 50 °C [26].

Special membranes for desalination by membrane distillation were obtained from a solution composed of poly(vinylidene fluoride-co-hexafluoropropylene) (PVDF-HFP) and PEG dissolved in N, N-dimethylacetamide (DMA). Optimal membranes were obtained under the following conditions: PVDF-HFP concentration 20% *w*/*w*, PEG concentration 6% *w*/*w*, air gap 25 cm, coagulation temperature 37.5 °C, and solution flow 19 mL/min [27].

Membranes were obtained from a solution of polylactic acid (PLA) in dichloromethane (DCM) and N, N-dimethylformamide (DMF) using two types of spinnerets, a nozzle and a channel, and their structure and tensile properties were compared. The use of a channel-type spinneret leads to a more porous membrane structure with a lower crystallization capacity than the structure of membranes passing through the nozzle spinneret [28].

#### 2.1.2. Polymer Solution and/or Bore Fluid Modifications

Another modification is changing the polymer solution and/or bore fluid. Pore-forming compounds such as polyvinylpyrrolidones (PVP) and polyethylene glycols (PEG) (which are pore formers), as well as other substances that change the membrane hydrophilicity are often added to the polymer solution [29,30,31,32,33,34,35,36,37,38,39,40,41,42,43,44,45,46,47,48,49,50,51,52,53,54,55,56,57,58,59,60,61,62,63,64]. The type of bore fluid affects the porosity of the internal skin layer or causes its “atrophy”. Below are publications discussing the effects of changes in the polymer solution and/or bore fluid on the properties of the membranes obtained.

Another publication compares polyetherimide and PES capillary membranes obtained under the same spinning conditions. PES membranes were more porous and showed a higher absorption flux than polyetherimide membranes, which was the result of their structure (large macropores and high surface porosity) [29].

**Figure 3:**
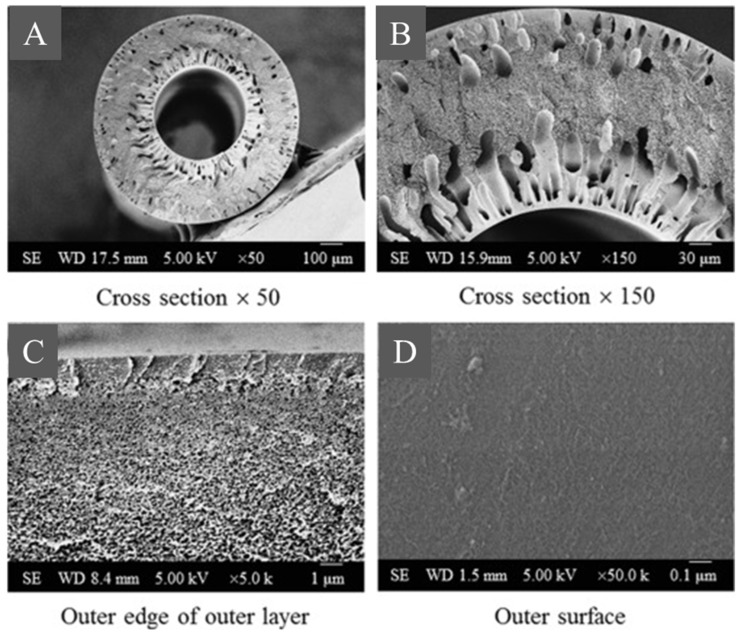
Sections of PMIA hollow fiber. The picture was modified from a previous article [30]. Scale bars: (**A**) 100 µm, (**B**) 30 µm, (**C**) 1 µm, (**D**) 0.1 µm.

In the next study, a poly(m-phenyleneisophthalamide) (PMIA) membrane was spun using the dry–wet method. The membrane was characterized by an asymmetric structure with diversified porosity in the cross-section (dense outer surface, spongy transition layer, and finger structure near the inner surface—Figure 3) [30].

A capillary PVDF membrane for the separation of dyes and sodium salts in textile wastewater was developed and produced. The membrane was characterized by a high separation efficiency of salts and dyes in textile wastewater. Moreover, the membrane showed good stability and anti-fouling properties during long-term operation with a mixture of salts and dyes [31].

Poly(lactide-co-glycolide) (PLGA) membranes obtained via the wet-spinning process were tested for structure and mechanical strength. It was found that with the increase in the concentration of PLGA in the membrane-forming solution, the structure of the membranes changed from a loose finger-like structure with a large number of macropores to a dense spongy one with micropores [32].

Six membrane-forming solutions with different types of PVDF were selected for the spinning experiments. The influence of the type of PVDF on the morphology and mechanical properties of the obtained capillaries were investigated. It was observed that the morphology, porosity, and mechanical properties of the membranes were decisively influenced by the concentration of PVDF in the membrane-forming solution [33].

Polyvinyl chloride (PVC) membranes for gas separation were obtained via the spinning method. The membrane dope solution was characterized by a high polymer content. The obtained membranes had a high selectivity and thin active skin layers. The authors claimed that this was the first publication to describe the successful use of PVC membranes for gas separation [34].

Capillary membranes were prepared from a solution containing PVDF polymer, DMF solvent, and PVP pore-forming agent. The core fluid was a mixture of DMF or ethanol in water. Membrane porosity was determined via the gravimetric method. The obtained membranes were characterized by up to 80% porosity, from a 0.12 to 0.27 µm average pore size and very good mechanical properties [35].

Capillary CA membranes intended for the forward osmosis (FO) process were obtained. They were then heat-treated to reduce the pore sizes of the membrane. These membranes had a retention rate of 90.2% for NaCl and 96.7% for MgCl_2_. As the concentration of the solution increased, the flux of the passing water increased. These results made it possible to predict the usefulness of membranes for FO processes [36].

Capillary PVDF membranes for gas separation were obtained. Orthophosphoric acid and lithium chloride monohydrate (LiCl × H_2_O) were used interchangeably as the pore formers. The membranes were characterized by a thin skin layer and a finger-spongy filling. Gas permeation tests showed a 33% higher CO_2_ flux and a larger mean pore size in the case of phosphoric acid being used as the pore-forming agent than in the case of using LiCl × H_2_O as the pore-forming agent [37].

Polyimide (PI) capillary membranes were obtained by modifying them with polyethyleneimide (PEI) dissolved in bore fluid. A controlled cross-linking reaction took place between the PI membrane and PEI. Depending on the amount of PEI in the bore fluid, two different types of membranes were obtained: one with a densely cross-linked gas-selective inner layer and the other one with fully cross-linked porous and selective membranes, effectively separating similarly sized proteins such as albumin (BSA) and hemoglobin [38].

Using a PVDF-HFP solution in NMP, capillary membranes were prepared with the addition of PEG as the pore-forming agent. The addition of LiCl to the spinning solution eliminated the formation of macropores. Increasing the temperature of the coagulation bath to 40 °C prevented irregular internal contours of the membrane. Membranes composed of PVDF-HFP/PEG/LiCl/NMP (15/3/3/79 by weight) showed a high permeability of pure water (PWP) and MWCO of 150 kDa [39].

The obtained three types of capillary membranes from CA, cellulose acetate butyrate (CAB), and cellulose acetate propionate (CAP) are characterized by similar PWP values, surface roughnesses, and zeta potentials. The hydrophilicity of the membranes increased in the CAP > CAB > CA order. The most optimal CA membrane with the highest hydrophilicity showed the best anti-fouling properties for humic acid and BSA [40].

The three-block copolymer Pluronic/poly(ethylene oxide) (PEO)/poly(propylene oxide) (PPO) was used to modify the surface of the obtained PES membranes. The PEO layer formed on the inner surface of the membrane improved the retention of dissolved substances. Membranes obtained with the addition of Pluronic showed high PWP values and an MWCO of 9 kDa [41].

Capillary PVDF membranes were obtained via the spinning process. LiCl at various concentrations was added to the membrane spinning solution. A highly effective porous surface was obtained at a low concentration of LiCl. As the salt concentration increased, the pore size, contact angle, and N_2_ permeability decreased. The highest flux of PWP was shown by the membrane with 5 wt. LiCl [42].

Capillary membranes for gas separation were prepared from a membrane dope solution consisting of poly(o-Anisidine) (POAn), NMP, and THF as the polymer solvents. The length of the air gap was chosen so as to obtain the skin layer of the membrane. The POAn membranes obtained with a large air gap had a compact structure conductive to good gas separation. Gases such as O_2_/N_2_, CO_2_/N_2_ and H_2_/N_2_ could be effectively separated on such a membrane [43].

Membranes for membrane distillation were made of PVDF composite and CaCO_3_-dispersed modified nanoparticles. LiCl and PEG were used as the pore-forming agents of the non-solvent. Such membranes had a good operating stability in continuous desalination experiments [44].

PVDF capillary membranes with excellent mechanical strengths and a very dense outer and inner skin layer were obtained from a PVDF/TEP spinning solution. To improve the hydrophilicity of the membranes for water and wastewater applications, two types of PEGs were used as the pore-forming agents, which significantly improved the PWP flow [45].

In preparing PSf capillary membranes, oil was added to the polymer solution, which acted as a hole-forming agent to obtain a hollow structure. These drop-like PSf membranes were characterized by increased mechanical stability [46].

Capillary PVDF membranes were produced for CO_2_ stripping via membrane contactors. Compounds increasing hydrophilicity, such as LiCl, glycerin, PEG-400, phosphoric acid, and methanol, were added to the membrane solution. The highest efficiency of the CO_2_ stripping process was achieved by the PVDF/PEG-400 membrane [47].

An ester-crosslinked capillary membrane was developed by changing the membrane dope solution and the parameters of the spinning process. The membranes obtained were characterized by a good separation efficiency and resistance to CO_2_ plasticization. A high selectivity for CO_2_/CH_4_ and CO_2_ permeability of 110 GPU were obtained [48].

**Figure 4:**
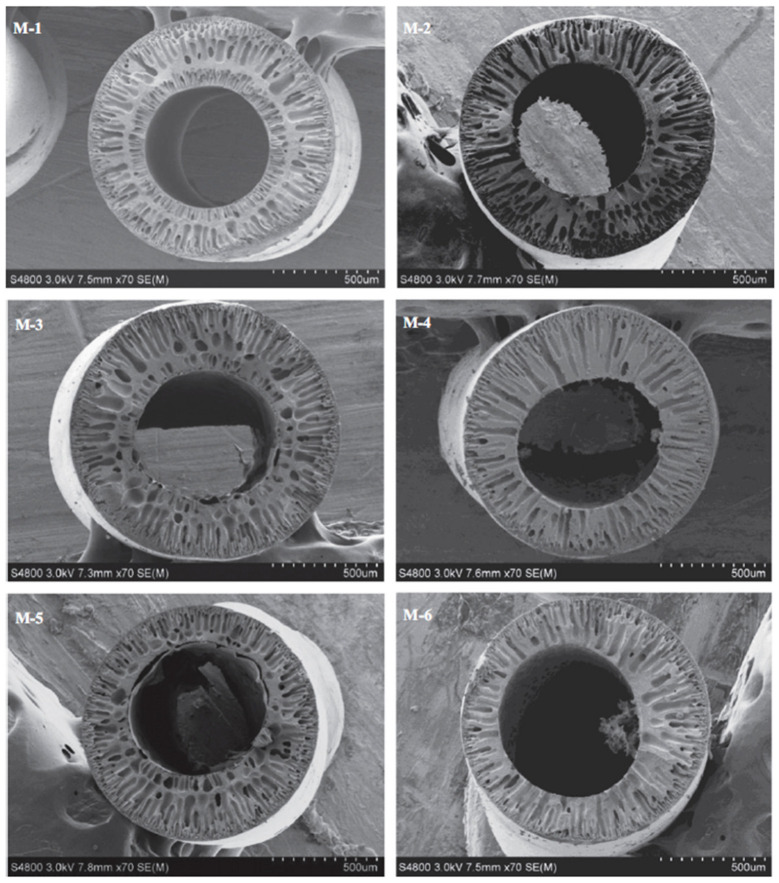
SEM images of cross-sections of PVB hollow-fiber membranes. The images were modified according to the literature [49]. Scale bars: 500 µm. The membranes of M-1, M-3 and M-5 were fabricated using 50 wt% ethanol aqueous solution as bore liquid, whereas the membranes of M-2, M-4 and M-6 were fabricated with 90 wt% NMP aqueous solution as bore liquid.

The changes in the capillary parameters of the obtained polyvinyl butyral/perfluorosulfonic acid PVB/PFSA membranes depending on the composition of the core fluid were investigated. For a 50% aqueous ethanol solution and a 90% aqueous NMP solution in the core fluid, the membranes had an asymmetric finger structure (Figure 4). Membranes with a higher PFSA content in the dope solution and 90% NMP in the core fluid had a higher UFC than membranes with a lower PFSA content and 50% ethanol in the core fluid [49].

Membranes were prepared from a dope solution composed of PSf/NMP/PEG-400/branched polyester (HBPE). The influence of HBPE content on the properties of the membrane was investigated. As the HBPE content increased from 0 to 3% by weight, the value of the contact angle decreased, and the porosity, the average pore size, the flow of PWF, and the breaking strength increased [50].

**Figure 5:**
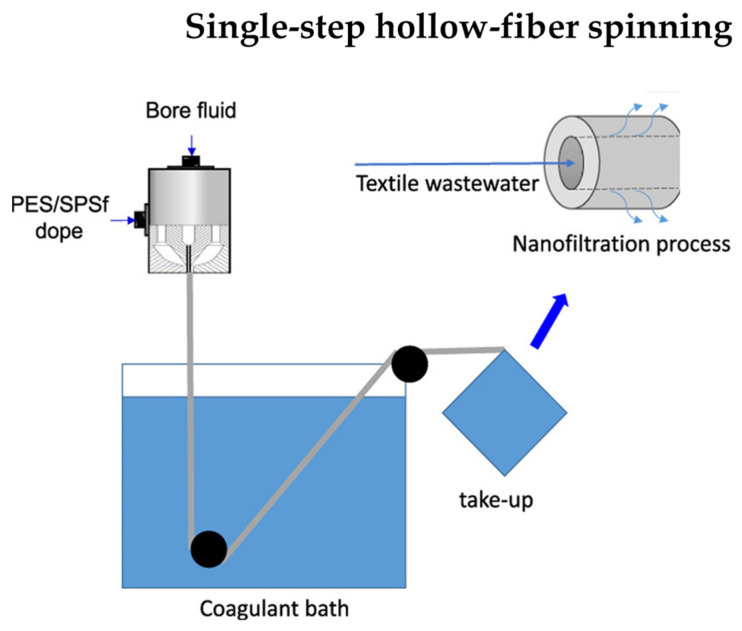
Schematic diagram of the PES membrane fabrication process. Schematic was modified from a previous article [51].

PES-modified capillary membranes were obtained for nanofiltration processes. Small amounts of sulfonated polysulfone (SPSf) were added to the spinning solution, and hydrophilic PEG and PVP were added to the core fluid (Figure 5). This way, the pore size was reduced, and the formation of finger macropores in the membrane structure was inhibited. The membrane modified in this way had a retention of 70% for 1 kDa PEG [51].

PES membranes modified with diphenylsulfone were prepared for gas separation. The resulting polytrimethylphenylene ether sulfone (TPES) capillary membranes had high O_2_ and CO_2_ permeabilities and very good gas separation efficiencies. The selectivity of O_2_/N_2_, CO_2_/N_2_, and CO/CH_4_ was very high. The new TPES membranes had a separation efficiency comparable to those of Matrimid [52].

Membranes spun from a PAN/dimethylsulfoxide (DMSO)/H_2_O solution were modified by adding Pluronic F127 to the dope solution. This resulted in faster solvent–non-solvent separation, resulting in a faster phase inversion process. Adding 3 wt.%. F127 to the dope solution significantly improved the filtration efficiency, improved the mechanical properties of the membranes, and increased the gas permeability [53].

Ionic liquids were used as the dope solution for capillary cellulose membranes. The polymer solutions contained acetates or phosphates as anions and cations based on imidazolium. The filtration efficiency was determined using dyes in a solution of water and ethanol. As a result of optimally selected spinning solutions, a cellulose-resistant membrane was obtained [54].

Active amino groups derived from the polyethyleneimine (PEI) used in the bore fluid were introduced into the blend of SPSf/PES capillary membranes. PEI enables the creation of more selective membranes by eliminating macrovoids from their structure. The obtained membranes had an average pore size of 0.57 nm and a MWCO of 157 Da. The retention rate for metals such as Ni^2+^, Zn^2+^, and Cu^2+^ was over 90% [55].

PVP was used as the pore-forming agent for the obtained capillary PVB membranes. As the concentration of the PVP increased, the flux first increased and then decreased. A low concentration of PVB resulted in a loose structure of the membranes, which increased the PWF, while an increase in the concentration of the PVP caused a decrease in the flow of PWF. The addition of DMA to the core fluid changed the inner surface of the membrane to a more porous one [56].

Capillary membranes were obtained from a dope solution composed of PVDF/DMA/LiCl/PEG-400, where LiCl and PEG-400 were used as the pore-forming agents. The membrane permeability increased with increases in its effective porosity. The synergistic action of LiCl/PEG-400 resulted in a high flow and good mechanical strength of the membranes [57].

Another study investigated the influence of the composition and flow velocity of the bore fluid on the parameters of capillary PVDF membranes. NMP was used as the polymer solvent, and water and PVP k-17 were used as the pore-forming agents. Apart from the bore fluid, the remaining parameters of the spinning process were unchanged. The wall thickness and porosity of the membranes were found to be dependent on the composition and flow velocity of the bore fluid. With increases in its flow rate, the internal diameter of the capillaries increased, and the thickness of their walls decreased [58].

PES capillary membranes were made and covered with a thin layer of polymer composite membranes. Four different water-phase monomers were used, piperazine (PIP), 1,3,5-benzenetrithol, m-phenylenediamine, and 1,3-benzenedithiol, while the monomer of the organic phase was trimesoyl chloride (TMC). The best results for water vapor permeability and water vapor/N_2_ selectivity were obtained using a membrane with 1,3-benzenedithiol monomer [59].

A PVDF-HFP/graphene oxide (GO)/ODS capillary membrane was made using a composition of 12 wt.% polymer and (0, 1, 3 and 5%) graphene oxide in NMP as the solvent. The resulting capillaries were silanized using ODS. The membranes after silanization showed a higher flux for water and a greater contact angle compared to the initial membranes [60].

Capillary membranes were made from a spinning solution composed of PAN and AgNO_3_ dissolved in DMF. PAN-Ag membranes modified with silver nanoparticles showed significant differences compared to PAN membranes. The PAN-Ag membranes had better mechanical properties, greater strengths and Young’s modulus values, as well as lower elongation than unmodified PAN membranes [61].

For the separation of oxybenzone and bisphenol A from water, special capillary composite membranes with the composition of PES/carboxylated graphene oxide/Cu_2_S were developed. The membranes were characterized by a high hydrophilicity, good mechanical strength, and thermal stability. They also showed high volumetric porosity [62].

High ultrafiltration (UFC) efficiency capillary membranes were obtained using a PVDF/CaCO_3_ spinneret solution in DMF as solvent. The best UFC performance was obtained by using 10% CH_3_COOH as the bore fluid and 1 M HCl in the second coagulation bath [63].

Two types of PMIA capillary membranes were made: one containing PEG and LiCl as pore formers, the other without additives. The PEG-containing membranes created dense spongy structures, which resulted in an increase in their hydrophilicity. The membranes with the highest PWF and retention rate for BSA (98%) were obtained from a composition containing PMIA/PEG 2 wt.%/LiCl 4 wt.% [64].

### 2.2. Preparation of Multi-Component Membranes

#### 2.2.1. Blend Membrane Fabrication

Other examples include two- or multi-component blend membranes. The two polymers are dissolved and mixed in solution. Most often, a hydrophilic polymer is added to the hydrophobic polymer to increase flux through the membrane [65,66,67,68,69,70,71,72,73,74,75,76,77]. 

A PES/terpolymer(poly(methyl methacrylate-acrylic acid-vinylpyrrolidone) blend capillary membrane was made usin DMA as the solvent. As the terpolymer content in the membrane increased, the flux and hydrophilicity of the membrane increased significantly, increasing the protein anti-fouling properties of the membrane [65].

A PSf/β-cyclodextrin (β-CD) blend capillary membrane was designed and manufactured to remove endocrine-disrupting substances such as di-(2-ethylhexyl)phthalate (DEHP) from water. The results of the research showed effective removal of DEPH from drinking water and good water permeability through the PSf/CD membrane modified in this way [66].

A pH-sensitive membrane was developed consisting of PES and poly(methyl co-4-vinylpyridine methyl acrylate) (P (MMA-4VPy)). The resulting PES/P (MMA-4VPy) blend capillary membrane had excellent pH sensitivity and pH reversibility. Moreover, the membrane had good Cu^2+^ ion adsorption properties [67].

New Matrimid^®^5218/PEG or PEO-PDMS-blend capillary membranes were developed for gas separation. It was observed that increasing the concentration of PEG in the membranes increased the CO_2_ permeability and reduced the plasticization pressure of CO_2_. On the other hand, the 12 wt.% content of PEO-PDMS in the membranes showed a twice higher CO_2_/N_2_ selectivity and improved the resistance of the capillaries to plasticization [68].

Another capillary gas separation blend membrane was obtained using a polydimethylsiloxane (PDMS) composition from PAN. The membrane showed very high O_2_ and CO_2_ permeability and very good O_2_/N_2_ and CO_2_/N_2_ selectivity [69].

**Figure 6:**
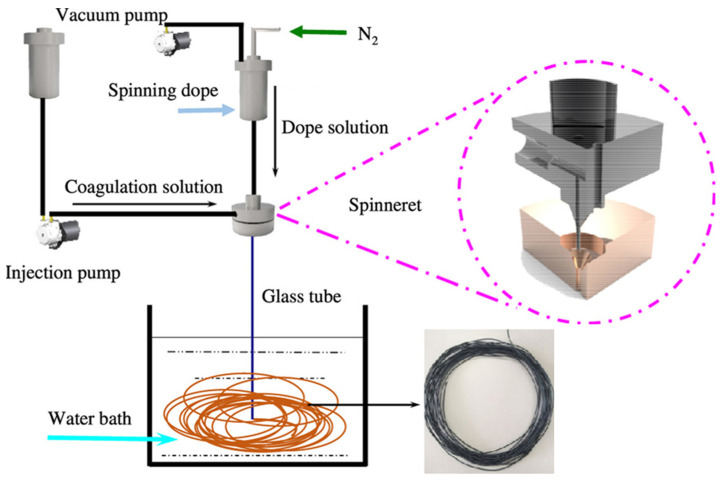
GO/PI membranes fabrication schematic diagram. Schematic was modified from a previous article [70].

GO/PI blend capillary membranes have been developed for the desalination of seawater (Figure 6). During the desalination process for GO/PI membranes, the salt retention rate was as high as 99.8% with a high water flow. Moreover, the membranes were characterized by a high durability and stability during long-term operation for seawater desalination [70].

**Figure 7:**
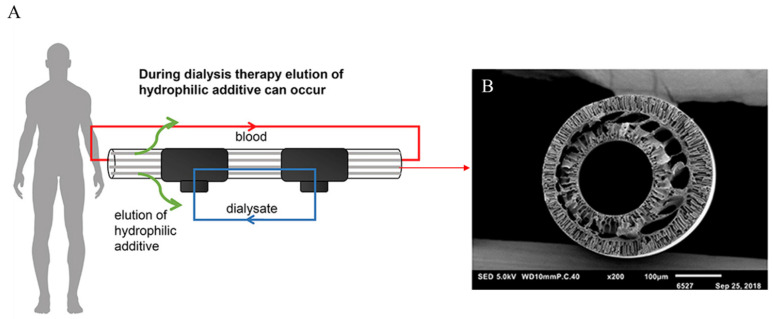
General schematic of dialysis therapy (**A**) and SEM image of PES-SlipSkin™ hollow-fiber membrane. Figure was prepared based on a previous article [71]. Scale bar: (**B**) 100 µm.

Special capillary membranes for long-term hemodialysis with the PES/SlipSkin™ (SS) composition were developed and manufactured (Figure 7). The membranes removed, to a significant degree, compounds such as indoxyl sulfate (IS), hippuric acid (HA), and, above all, creatinine. Moreover, they had very good blood compatibility and fouling resistance [71].

PVDF/polyvinyl alcohol (PVA)-blend membranes were developed to improve their hydrophilicity. An increase in the PVA content in membranes caused an increase in PWF, while the retention factor decreased. Moreover, PVA improved the anti-fouling properties of the membranes [72].

A PVC/polystyrene (PSR)-blend capillary membrane was developed using DMA as the solvent. As the PSR content in the PVC/PSR membrane increased, the retention for PVP increased, while the PWF value did not change [73].

A mixed PES/poly(styrene-alane-maleic anhydride) (PSMA) membrane was developed and prepared. The PES/PSMA-H carboxylic membrane was obtained under the action of NaOH solution, which showed a good sensitivity to pH [74]. 

New PANI/PSf/PVP-blend capillary membranes were developed and made via a wet–dry spinning technique. With increases in the PANI content in the membranes, the PWF, retention, anti-fouling properties, and thermal resistance increased. On the other hand, the contact angle decreased, which proved the increase in hydrophilicity of the obtained membranes [75].

For comparative purposes, PVDF and PVDF-co-chlorotrifluoroethylene (CFTE) membranes were made. Both membranes were treated with a strongly alkaline solution of 1 M NaOH + NaOCl in order to evaluate the physical and mechanical resistance. The mechanical properties of the membranes were reduced by 90% for PVDF and only 10% for PVDF-co-CFTE compared to the original membranes [76].

Mixed PVPF/PMMA capillary membranes were made using PVP as the pore-forming agent and a DMF/DMA mixture as the polymer solvent. For PVPF/PMMA/PVP/DMA/DMF concentrations of 18/0.5/1.5/40/40 wt.%, respectively, the highest PWF flow was achieved, and the highest BSA retention amounted to 73% [77].

#### 2.2.2. Degradable-Blend Membranes

A specific variety of blend membranes are degradable membranes, where one of them disintegrates during work, causing an increase in porosity, which leads to an increase in flux [78,79,80].

The method of preparation of PSf/PUR [78,79] and PSf/CA [80] partly degradable-blend hollow-fiber membranes was presented. The membranes built of PSf stable polymers and PUR or CA polymers containing degradable ester groups were obtained via the dry–wet spinning phase-inversion technique and then treated with a NaOH solution using the flowing method. The membrane morphology was more porous, and UFCs were higher after the hydrolysis process. Using degradable polyesters in PSf/PUR and PSf/CA membranes allowed the researchers to achieve slow, partial removal PUR and CA during the membrane’s long operation time in water environment. The gradual process of porous clogging should balance the slow removal of PUR and CA ester group decomposition products from the membrane, reducing the fouling process, which should lead to opening of new porous. These membrane properties allowed the researchers to retain efficient membrane processes for a longer time, despite the fouling progress. 

#### 2.2.3. Dual-Layer Membranes

In dual-layer membranes, two polymers are dissolved separately in two tanks and introduced into a triple-orifice spinneret. In such membranes, one layer comprises its scaffolding, and the other is a separation layer [81,82,83,84,85,86,87,88,89,90,91].

**Figure 8:**
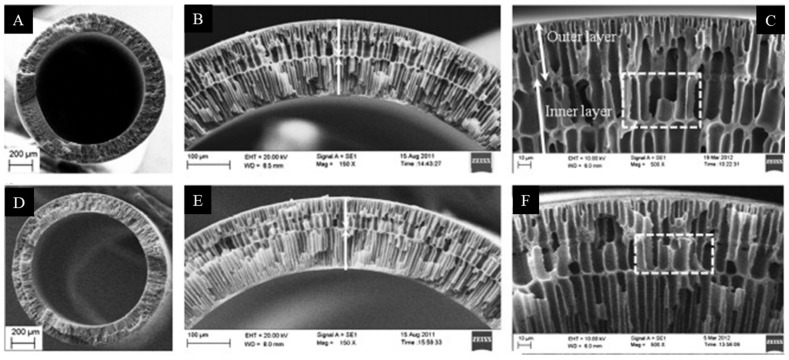
SEM image of cross-sections of PAI/PES dual-layer hollow fibers. Figure was modified from a previous article [81]. Scale bars: (**A**,**D**) 200 µm, (**B**,**E**) 100 µm, and (**C**,**F**) 10 µm.

A novel double-layer PAI/PES capillary membrane (PAI-outer layer, PES-inner layer) for forward osmosis (FO) processes was obtained by using a triple-orifice spinneret. Then, the outer layer of PAI was covered with a thin layer of PEI polyelectrolyte (Figure 8). The membrane obtained in this way showed very good performance in FO processes [81].

Special double-layer capillary membranes were obtained using a three-nozzle spinneret for ethanol dehydration in the pervaporation process. The membrane had an outer support layer and an inner selective layer and was characterized by a high PWF and good water/ethanol selectivity [82].

**Figure 9:**
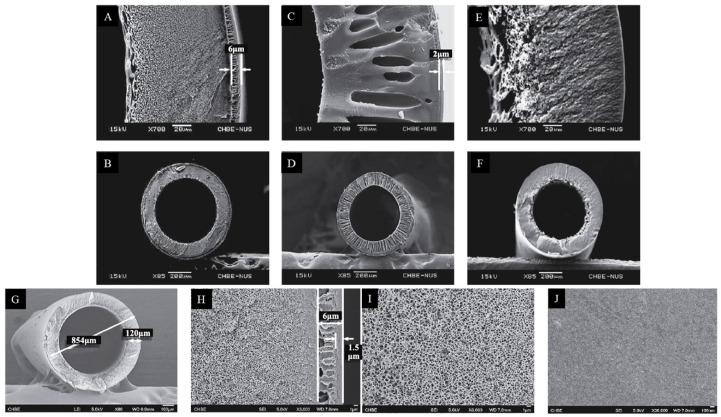
SEM images of the cross-sections for different dual-layer hollow-fiber membranes: (**A**,**B**) PBI/PES; (**C**,**D**) PBI/P84; (**E**,**F**) PBI/PAN. The structure of the PBI/PES dual-layer NF hollow-fiber membranes: (**G**) cross section, (**H**) magnification of cross section, (**I**) inner-layer inner surface, and (**J**) outer-layer outer surface. Scale bars: (**A**,**C**,**E**) 20 µm; (**B**,**D**,**F**) 200 µm; (**G**,**J**) 100 µm; (**H**,**I**) 1 µm. Picture was modified from a previous article [83].

A dual-layer polybenzimidazole (PBI)/PES-PVP capillary membrane designed for NF processes was developed and manufactured. The selective outer layer was PBI with excellent chemical resistance. The inner support layer was PES/PVP with a low cost, high hydrophilicity, and high porosity, as well as good mechanical properties (Figure 9). The PBI/PES-PVP membrane had high salt retention values, a narrow pore size distribution, and an MWCO of 249 Da [83].

Dual-layer PVDF capillary membranes for the vacuum membrane distillation (VMD) process were made. The membranes were characterized by a high porosity and permeability as well as good strength. Their application allowed the VMD flux to be enhanced by 54% [84].

**Figure 10:**
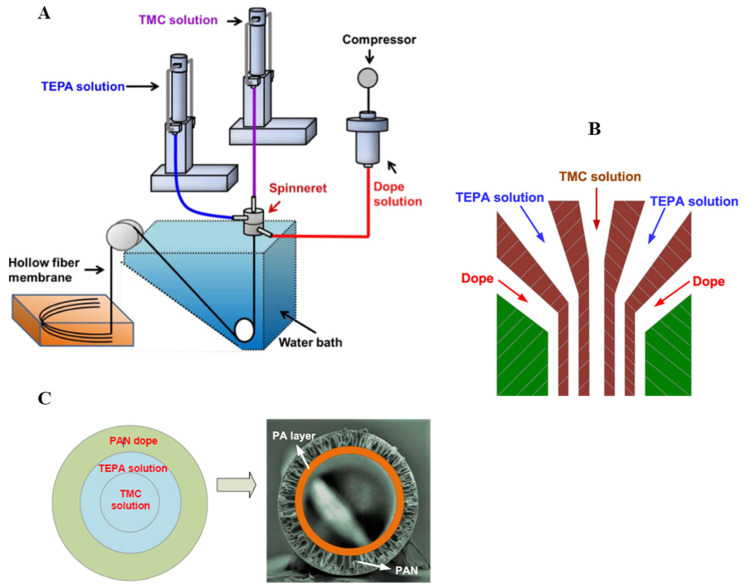
Schematic diagram od PA/PAN dual-layer membrane fabrication and SEM image of membrane cross sections. (**A**) PA/PAN composite hollow-fiber membrane fabrication frame, (**B**) profile of the triple orifice spinneret, and (**C**) PA layer formation. Figure was prepared according to the literature [85].

A triple-orifice spinneret was used to obtain a polyamide (PA)/PAN dual-layer capillary membrane, where a PAN solution was fed to the outer nozzle, tetraethylene pentamine (TEPA) was fed to the middle nozzle, and a tetramethylsilane (TMC) solution was fed to the inner nozzle (Figure 10). Then, the inner layer of the membrane was covered with a layer of PA. SiO_2_ was added to the spinning solution to increase its hydrophilicity [85].

A dual-layer capillary membrane was made for use in FO, where both layers consisted of PES. A dense inner layer and a porous loose outer layer were obtained via co-extrusion on a three-nozzle spinneret. The dense inner layer retained organic foulants greater than 300 Da [86].

An innovative dual-layer capillary membrane was made of sulfonated polyphenylsulfone (sPPSU) and PBI forming the outer selective layer and PSf, which constituted the inner support layer. The sPPSU acted as an ionic cross-linker with the PBI. The obtained PBI-sPPSU/PSf membrane may be helpful in purifying H_2_ and CO_2_ at elevated temperatures [87].

A multilayer capillary membrane for NF organic solvents was developed. The support layer was made of monomers such as PEI, PIP, and TMC. The inner thin active layer was PA. The membranes were characterized by a very good NF efficiency at a low working pressure of 2 bar, which is advantageous in practical applications [2].

Dual-layer microporous hollow-fiber membranes were manufactured using Torlon PAI as the material of the selective outer layer and PES as the material of the porous supportive inner layer. A positively charged selective layer was developed via simple polyelectrolyte cross-linking using polyallylamine (PAAm). The newly developed (PAI/PAAm)/PES dual-layer membranes showed a salt-water permeability of 15.8 L/m^2^ h bar and high Mg^2+^ and Ca^2+^ rejections of 94.2% and 92.3%, respectively, at an operating pressure of 2 bar using a 3000 ppm TDS feed solution [88].

PAI and PES were used to make dual-layer capillary membranes. The selective outer layer was PAI, while the porous inner carrier layer was PES. Subsequently, the outer layer of the PEI was modified to produce nanofiltration skin by crosslinking and depositing polyelectrolytes on it. Finally, membranes with the following parameters were obtained: PWP: 4.1 L/m^2^ × h × bar; retention for MgCl_2_: 97%. In the FO process, the membranes showed a water flux ranging from 21 to 39 L/m^2^ × h [89].

Dual-layer composite membranes were obtained, where the main component in both layers was PVDF. In the inner layer, PVA polymers of various amounts were added to the PVDF in order to increase its hydrophilicity. With a lower PVA content in the inner layer, higher retention was achieved. For the membrane without PVA in the outer layer and the use of water as both an external and internal coagulant, low flux and low retention values were noted. The addition of ethanol to both coagulant solutions significantly improved the flux and retention efficiency [90].

### 2.3. Corrugated Membranes

Membranes featuring corrugated surfaces represent another illustrative case. Methods for obtaining membranes with a developed inner and/or outer surface are presented below. One of the methods of their production is the special design of the spinneret with a corrugated nozzle. Another way is to set the flow rate of the polymer solution and the pressure of the core fluid [91,92,93].

A method of obtaining hollow-fiber membranes with a developed inner wall structure has been established by setting appropriate spinning conditions. Obtaining a developed surface of membranes without interference into the spinneret structure is feasible. Membranes with a developed inner surface have a higher water flux than that of membranes with a smooth surface made from the same membrane-forming solution [91].

Capillary membranes with an external corrugated structure were obtained from a PES/PVP solution. They were spun with slow solidification of the polymer, which caused the formation of a skin layer with different thicknesses and its folding. Externally corrugated membranes were obtained, and for comparison purposes, membranes spun under the same conditions with a smooth surface were obtained as well. The membranes had comparable MWCO values, pore size distributions, and wall thicknesses. In contrast, the PWP of externally corrugated membranes increased [92].

**Figure 11:**
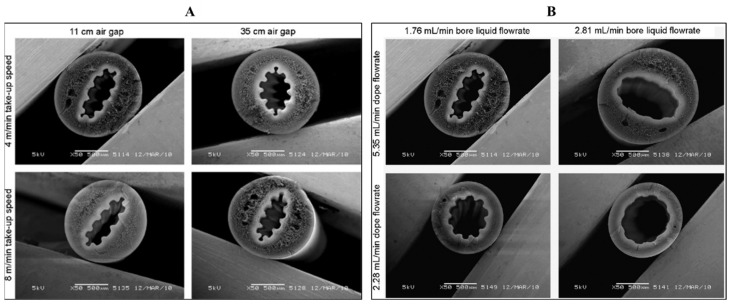
SEM images of cross-sections of PES/PVP corrugated membranes fabricated using (**A**) a dope flowrate of 5.35 mL/min, a bore liquid flowrate of 1.76 mL/min with varying take-up speeds or air gaps; (**B**) a take-up speed of 4 m/min, air gap of 11 cm, and varying dope or bore liquid flowrates. Scale bars: 500 µm. This figure was modified from the literature [93].

Using a PES/PVP solution, using a special spinneret with a microstructured needle, capillary membranes with a corrugated inner surface were obtained (Figure 11). For comparison, smooth membranes were obtained using a normal spinneret spun under the same conditions. It was found that the internal PWP for both types of membranes was similar. It was also found that the corrugated membranes had larger pores in the skin layer than the smooth membranes [93].

## 3. Conclusions

This paper presents 77 of the most important techniques for obtaining polymeric hollow-fiber membranes that have been published in the last 20 years in renowned membrane publishers. Of all the membrane publications, the production of hollow-fiber membranes accounts for only a small percent. The authors usually focus on the use of ready-made membranes in various membrane processes. An important barrier to obtaining hollow-fiber membranes is the need to have a special installation described by the authors in this paper. Therefore, most publications concern the preparation of flat membranes, which are obtained in a simple way. However, hollow-fiber membranes, rather than flat membranes, are commonly used in membrane processes. Hollow-fiber membranes are more advantageous for the fabrication of membrane modules in industry because of their high membrane area to module volume ratios.

This paper presents methods of obtaining polymer capillary membranes via solution spinning. In the membrane-spinning installation, we can change several key parameters during the process, such as the speed of polymer extrusion from the spinneret and the speed of the take-up (winding) wheel. Both of these parameters must be synchronized with each other. An increase in the extrusion speed forces an increase in the removal speed, which increases the efficiency of the process, which is especially desired in industrial production. However, an increase in the pressure of bore fluid administration causes a higher capillary outflow velocity, but at the same time, the diameter of the membrane increases and the thickness of its walls decreases. The situation is similar with the air gap—when we increase it, the outflow velocity increases and the thickness of the capillary walls decreases, but their diameter does not increase. By changing the above-mentioned parameters, we can freely change the geometric parameters of the capillaries and the efficiency of the spinning process.

Modification methods for the preparation of the hollow-fiber membranes developed so far show that the potential of new techniques has not yet been exploited. This applies to dual-layer membranes, where we can use new polymers in the active layer, whereas single membranes due to poor mechanical parameters or high material costs could not be used before. The development of degradable membranes is another challenge. Polymer membranes, like other plastic products after a period of exploitation, pose a serious environmental problem as non-degradable waste. Therefore, degradable polymers for the production of hollow-fiber membranes are sought. Degradable polyurethanes, polylactides, and other polyesters, used alone as well as in blends or dual-layer membranes, have been successfully used.

The development of new techniques for obtaining membranes results in new membranes and their modifications, resulting in membranes with new transport/separation parameters, which significantly expands their application possibilities. Therefore, supporting researchers looking for new techniques for obtaining hollow fiber polymer membranes is a desirable direction.

## References

[1] Ferrera E., Ruigómez I., Vera L. (2024). Pilot scale application of a rotating hollow fibre membrane for direct membrane filtration of domestic wastewater. J. Water Process. Eng..

[2] Goh K.S., Chong J.Y., Chen Y., Fang W., Bae T.-H., Wang R. (2020). Thin-film composite hollow fibre membrane for low pressure organic solvent nanofiltration. J. Membr. Sci..

[3] Jonkers W.A., Cornelissen E.R., de Vos W.M. (2023). Hollow fiber nanofiltration: From lab-scale research to full-scale applications. J. Membr. Sci..

[4] Sikorska W., Wasyłeczko M., Przytulska M., Wojciechowski C., Rokicki G., Chwojnowski A. (2021). Chemical degradation of psf-pur blend hollow fiber membranes—Assessment of changes in properties and morphology after hydrolysis. Membranes.

[5] Sikorska W., Przytulska M., Wasyłeczko M., Wojciechowski C., Kulikowski J.L., Chwojnowski A. (2021). Influence of hydrolysis, solvent and PVP addition on the porosity of psf/pur blend partly degradable hollow fiber membranes evaluated using the MeMoExplorer software. Desalination Water Treat..

[6] Chwojnowski A., Wojciechowski C., Dudziński K., Łukowska E. (2009). Polysulphone and polyethersulphone hollow fiber membranes with developed inner surface as material for bio-medical applications. Biocybern. Biomed. Eng..

[7] Unger R., Huang Q., Peters K., Protzer D., Paul D., Kirkpatrick C. (2005). Growth of human cells on polyethersulfone (PES) hollow fiber membranes. Biomaterials.

[8] Fukuda M., Saomoto H., Shimizu T., Namekawa K., Sakai K. (2019). Observation and proposed measurements of three-dimensional tortuous capillary pores with depth for hollow fiber hemoconcentrator membrane using dynamic force microscopy. Adv. Biomed. Eng..

[9] PES Hollow Fiber Dialyzer (High-Flux Series). https://www.peony-medical.com.

[10] Said N., Lau W.J., Ho Y.-C., Lim S.K., Abidin M.N.Z., Ismail A.F. (2021). A review of commercial developments and recent laboratory research of dialyzers and membranes for hemodialysis application. Membranes.

[11] Ahmed H.M.M., Salerno S., Piscioneri A., Khakpour S., Giorno L., De Bartolo L. (2017). Human liver microtissue spheroids in hollow fiber membrane bioreactor. Colloids Surf. B Biointerfaces.

[12] Wasyłeczko M., Wojciechowski C., Chwojnowski A. (2024). Polyethersulfone Polymer for Biomedical Applications and Biotechnology. Int. J. Mol. Sci..

[13] Wasyłeczko M., Sikorska W., Chwojnowski A. (2020). Review of synthetic and hybrid scaffolds in cartilage tissue engineering. Membranes.

[14] Modi A., Verma S.K., Bellare J. (2018). Hydrophilic ZIF-8 decorated GO nanosheets improve biocompatibility and separation performance of polyethersulfone hollow fiber membranes: A potential membrane material for bioartificial liver application. Mater. Sci. Eng. C.

[15] Khulbe K.C., Matsuura T. (2016). Recent progress in polymeric hollow-fibre membrane preparation and applications. Membr. Technol..

[16] Sikorska W., Milner-Krawczyk M., Wasyłeczko M., Wojciechowski C., Chwojnowski A. (2021). Biodegradation process of psf-pur blend hollow fiber membranes using *escherichia coli* bacteria—Evaluation of changes in properties and porosity. Polymers.

[17] Matveev D.N., Anokhina T.S., Volkov V.V., Borisov I.L., Volkov A.V. (2023). Fabrication of Hollow Fiber Membranes: Effect of Process Parameters (Review). Membr. Membr. Technol..

[18] Hasbullah H., Kumbharkar S., Ismail A., Li K. (2011). Preparation of polyaniline asymmetric hollow fiber membranes and investigation towards gas separation performance. J. Membr. Sci..

[19] Liu H., Xiao C., Hu X., Liu M. (2013). Post-treatment effect on morphology and performance of polyurethane-based hollow fiber membranes through melt-spinning method. J. Membr. Sci..

[20] Loh C.H., Wang R. (2014). Fabrication of PVDF hollow fiber membranes: Effects of low-concentration Pluronic and spinning conditions. J. Membr. Sci..

[21] Deng K., Liu Z., Luo F., Xie R., He X.-H., Jiang M.-Y., Ju X.-J., Wang W., Chu L.-Y. (2017). Controllable fabrication of polyethersulfone hollow fiber membranes with a facile double co-axial microfluidic device. J. Membr. Sci..

[22] Li Y., Cao B., Li P. (2017). Fabrication of PMDA-ODA hollow fibers with regular cross-section morphologies and study on the formation mechanism. J. Membr. Sci..

[23] Yucel H., Culfaz-Emecen P.Z. (2018). Helical hollow fibers via rope coiling: Effect of spinning conditions on geometry and membrane morphology. J. Membr. Sci..

[24] Zhang B., Lu C., Liu Y., Zhou P., Yu Z., Yuan S. (2019). Wet spun polyacrylonitrile-based hollow-mesoporous fibers with different draw ratios. Polymer.

[25] Radjabian M., Koll J., Buhr K., Handge U.A., Abetz V. (2013). Hollow fiber spinning of block copolymers: Influence of spinning conditions on morphological properties. Polymer.

[26] Liu M., Xiao C., Hu X. (2011). Optimization of polyurethane-based hollow fiber membranes morphology and performance by post-treatment methods. Desalination.

[27] Khayet M., Cojocaru C., Essalhi M., García-Payo M., Arribas P., García-Fernández L. (2012). Hollow fiber spinning experimental design and analysis of defects for fabrication of optimized membranes for membrane distillation. Desalination.

[28] Li Y., Lim C.T., Kotaki M. (2015). Study on structural and mechanical properties of porous PLA nanofibers electrospun by chan-nel-based electrospinning system. Polymer.

[29] Bakeri G., Ismail A., DashtArzhandi M.R., Matsuura T. (2014). Porous PES and PEI hollow fiber membranes in a gas–liquid contacting process—A comparative study. J. Membr. Sci..

[30] Wang T., Zhao C., Li P., Li Y., Wang J. (2015). Fabrication of novel poly(m-phenylene isophthalamide) hollow fiber nanofiltration membrane for effective removal of trace amount perfluorooctane sulfonate from water. J. Membr. Sci..

[31] Liu H., Chen Y., Zhang K., Wang C., Zhang Y. (2019). Poly(vinylidene fluoride) hollow fiber membrane for high-efficiency sep-aration of dyes-salts. J. Membr. Sci..

[32] Wang Z., Lin J., Zhang D., Xun B., Yin J., Qian J., Dai G., Zhang N., Weng X., Huang Y. (2019). Porous morphology and mechanical properties of poly(lactide-co-glycolide) hollow fiber membranes governed by ternary-phase inversion. J. Membr. Sci..

[33] Figoli A., Simone S., Criscuoli A., Al-Jlil S., Al Shabouna F., Al-Romaih H., Di Nicolò E., Al-Harbi O., Drioli E. (2014). Hollow fibers for seawater desalination from blends of PVDF with different molecular weights: Morphology, properties and VMD performance. Polymer.

[34] Jones C., Gordeyev S., Shilton S. (2011). Poly(vinyl chloride) (PVC) hollow fibre membranes for gas separation. Polymer.

[35] Simone S., Figoli A., Criscuoli A., Carnevale M.C., Rosselli A., Drioli E. (2010). Preparation of hollow fibre membranes from PVDF/PVP blends and their application in VMD. J. Membr. Sci..

[36] Su J., Yang Q., Teo J.F., Chung T.-S. (2010). Cellulose acetate nanofiltration hollow fiber membranes for forward osmosis processes. J. Membr. Sci..

[37] Mansourizadeh A., Ismail A., Abdullah M., Ng B. (2010). Preparation of polyvinylidene fluoride hollow fiber membranes for CO_2_ absorption using phase-inversion promoter additives. J. Membr. Sci..

[38] Kopeć K., Dutczak S., Wessling M., Stamatialis D. (2011). Chemistry in a spinneret—On the interplay of crosslinking and phase inversion during spinning of novel hollow fiber membranes. J. Membr. Sci..

[39] Wongchitphimon S., Wang R., Jiraratananon R., Shi L., Loh C.H. (2011). Effect of polyethylene glycol (PEG) as an additive on the fabrication of polyvinylidene fluoride-co-hexafluropropylene (PVDF-HFP) asymmetric microporous hollow fiber membranes. J. Membr. Sci..

[40] Shibutani T., Kitaura T., Ohmukai Y., Maruyama T., Nakatsuka S., Watabe T., Matsuyama H. (2011). Membrane fouling properties of hollow fiber membranes prepared from cellulose acetate derivatives. J. Membr. Sci..

[41] Loh C.H., Wang R., Shi L., Fane A.G. (2011). Fabrication of high performance polyethersulfone UF hollow fiber membranes using amphiphilic Pluronic block copolymers as pore-forming additives. J. Membr. Sci..

[42] Naim R., Ismail A., Mansourizadeh A. (2012). Preparation of microporous PVDF hollow fiber membrane contactors for CO_2_ stripping from diethanolamine solution. J. Membr. Sci..

[43] Hasbullah H., Kumbharkar S., Ismail A., Li K. (2012). Asymmetric hollow fibre membranes based on ring-substituted polyaniline and investigation towards its gas transport properties. J. Membr. Sci..

[44] Hou D., Wang J., Sun X., Ji Z., Luan Z. (2012). Preparation and properties of PVDF composite hollow fiber membranes for de-salination through direct contact membrane distillation. J. Membr. Sci..

[45] Abed M.M., Kumbharkar S., Groth A.M., Li K. (2012). Ultrafiltration PVDF hollow fibre membranes with interconnected bicontinuous structures produced via a single-step phase inversion technique. J. Membr. Sci..

[46] Yao J., Wang K., Ren M., Liu J.Z., Wang H. (2012). Phase inversion spinning of ultrafine hollow fiber membranes through a single orifice spinneret. J. Membr. Sci..

[47] Naim R., Ismail A., Mansourizadeh A. (2012). Effect of non-solvent additives on the structure and performance of PVDF hollow fiber membrane contactor for CO_2_ stripping. J. Membr. Sci..

[48] Ma C., Koros W.J. (2012). High-performance ester-crosslinked hollow fiber membranes for natural gas separations. J. Membr. Sci..

[49] Lang W.-Z., Shen J.-P., Zhang Y.-X., Yu Y.-H., Guo Y.-J., Liu C.-X. (2013). Preparation and characterizations of charged poly(vinyl butyral) hollow fiber ultrafiltration membranes with perfluorosulfonic acid as additive. J. Membr. Sci..

[50] Zhao L.-B., Xu Z.-L., Liu M., Wei Y.-M. (2014). Preparation and characterization of PSf hollow fiber membrane from PSf–HBPE–PEG400–NMP dope solution. J. Membr. Sci..

[51] Gao J., Thong Z., Wang K.Y., Chung T.-S. (2017). Fabrication of loose inner-selective polyethersulfone (PES) hollow fibers by one-step spinning process for nanofiltration (NF) of textile dyes. J. Membr. Sci..

[52] Yong W.F., Chung N.T.-S., Weber M., Maletzko C. (2018). New polyethersulfone (PESU) hollow fiber membranes for CO_2_ capture. J. Membr. Sci..

[53] Wang L.-Y., Yu L.E., Lai J.-Y., Chung T.-S. (2019). Effects of Pluronic F127 on phase inversion and membrane formation of PAN hollow fibers for air filtration. J. Membr. Sci..

[54] Falca G., Musteata V.-E., Behzad A.R., Chisca S., Nunes S.P. (2019). Cellulose hollow fibers for organic resistant nanofiltration. J. Membr. Sci..

[55] Gao J., Wang K.Y., Chung T.-S. (2020). Design of nanofiltration (NF) hollow fiber membranes made from functionalized bore fluids containing polyethyleneimine (PEI) for heavy metal removal. J. Membr. Sci..

[56] Zhang P., Wang Y., Xu Z., Yang H. (2011). Preparation of poly(vinyl butyral) hollow fiber ultrafiltration membrane via wet-spinning method using PVP as additive. Desalination.

[57] Tang Y., Li N., Liu A., Ding S., Yi C., Liu H. (2012). Effect of spinning conditions on the structure and performance of hydrophobic PVDF hollow fiber membranes for membrane distillation. Desalination.

[58] Simone S., Figoli A., Criscuoli A., Carnevale M., Alfadul S., Al-Romaih H., Al Shabouna F., Al-Harbi O., Drioli E. (2014). Effect of selected spinning parameters on PVDF hollow fiber morphology for potential application in desalination by VMD. Desalination.

[59] Ingole P.G., Kil Choi W., Lee G.B., Lee H.K. (2017). Thin-film-composite hollow-fiber membranes for water vapor separation. Desalination.

[60] Dastbaz A., Karimi-Sabet J., Ahadi H., Amini Y. (2017). Preparation and characterization of novel modified PVDF-HFP/GO/ODS composite hollow fiber membrane for Caspian Sea water desalination. Desalination.

[61] Karbownik I., Fiedot M., Rac O., Suchorska-Woźniak P., Rybicki T., Teterycz H. (2015). Effect of doping polyacrylonitrile fibers on their structural and mechanical properties. Polymer.

[62] Modi A., Bellare J. (2019). Copper sulfide nanoparticles/carboxylated graphene oxide nanosheets blended polyethersulfone hollow fiber membranes: Development and characterization for efficient separation of oxybenzone and bisphenol A from water. Polymer.

[63] Huang D.-X., Wang L., Meng X.-R., Wang X.-D., Zhao L. (2013). Preparation of PVDF hollow fiber ultrafiltration membrane via phase inversion/chemical treatment method. Desalination Water Treat..

[64] Jiang Q., Zhang K. (2019). Preparation and characterization of high-flux poly(m-phenylene isophthalamide) (PMIA) hollow fiber ultrafiltration membrane. Desalination Water Treat..

[65] Zou W., Huang Y., Luo J., Liu J., Zhao C. (2010). Poly(methyl methacrylate–acrylic acid–vinyl pyrrolidone) terpolymer modified polyethersulfone hollow fiber membrane with pH sensitivity and protein antifouling property. J. Membr. Sci..

[66] Choi S.H., Chung J.W., Priestley R.D., Kwak S.-Y. (2012). Functionalization of polysulfone hollow fiber membranes with am-phiphilic β-cyclodextrin and their applications for the removal of endocrine disrupting plasticizer. J. Membr. Sci..

[67] Wang R., Xiang T., Yue W., Li H., Zhao C. (2012). Preparation and characterization of pH-sensitive polyethersulfone hollow fiber membranes modified by poly(methyl methylacrylate-co-4-vinyl pyridine) copolymer. J. Membr. Sci..

[68] Hu T., Dong G., Li H., Chen V. (2014). Effect of PEG and PEO−PDMS copolymer additives on the structure and performance of Matrimid^®^ hollow fibers for CO_2_ separation. J. Membr. Sci..

[69] Liang C.Z., Yong W.F., Chung T.-S. (2017). High-performance composite hollow fiber membrane for flue gas and air separations. J. Membr. Sci..

[70] Huang A., Feng B. (2018). Synthesis of novel graphene oxide-polyimide hollow fiber membranes for seawater desalination. J. Membr. Sci..

[71] Ter Beek O.E.M., Pavlenko D., Stamatialis D. (2020). Hollow fiber membranes for long-term hemodialysis based on polyethersul-fone-SlipSkin™ polymer blends. J. Membr. Sci..

[72] Li N., Xiao C., An S., Hu X. (2010). Preparation and properties of PVDF/PVA hollow fiber membranes. Desalination.

[73] Alsalhy Q.F. (2012). Hollow fiber ultrafiltration membranes prepared from blends of poly(vinyl chloride) and polystyrene. Desalination.

[74] Xiang T., Tang M., Liu Y., Li H., Li L., Cao W., Sun S., Zhao C. (2012). Preparation and characterization of modified polyethersulfone hollow fiber membranes by blending poly(styrene-alt-maleic anhydride). Desalination.

[75] Kajekar A.J., Dodamani B.M., Isloor A.M., Karim Z.A., Shilton S.J. (2015). Preparation and characterization of novel PSf/PVP/PANI-nanofiber nanocomposite hollow fiber ultrafiltration membranes and their possible applications for haz-ardous dye rejection. Desalination.

[76] Karkhanechi H., Vaselbehagh M., Jeon S., Shaikh A.R. (2018). Preparation and characterization of polyvinylidenedifluo-ride-co-chlorotrifluoroethylene hollow fiber membranes with high alkaline resistance. Polymer.

[77] Tan J.-S., Liu X.-M., Yu B., Cong H.-L., Yuan H., Yang S.-J., Li Z.-J., Gu C. (2014). Preparation of PVDF/PMMA Blend Hollow Fiber Ultrafiltration Membranes via Wet Spinning Method. Integr. Ferroelectr..

[78] Wojciechowski C., Chwojnowski A., Granicka L., Łukowska E., Grzeczkowicz M. (2016). Polysulfone/polyurethane blend de-gradable hollow fiber membranes preparation and transport–separation properties evaluation. Desal. Water Treat..

[79] Sikorska W., Wojciechowski C., Przytulska M., Rokicki G., Wasyleczko M., Kulikowski J.L., Chwojnowski A. (2018). Polysulfone–polyurethane (PSF-PUR) blend partly degradable hollow fiber membranes: Preparation, characterization, and computer image analysis. Desalination Water Treat..

[80] Wojciechowski C., Chwojnowski A., Granicka L., Łukowska E. (2017). Polysulfone/cellulose acetate blend semi degradable capillary membranes preparation and characterization. Desalination Water Treat..

[81] Setiawan L., Wang R., Shi L., Li K., Fane A.G. (2012). Novel dual-layer hollow fiber membranes applied for forward osmosis process. J. Membr. Sci..

[82] Ong Y.K., Chung T.-S. (2012). High performance dual-layer hollow fiber fabricated via novel immiscibility induced phase separation (I2PS) process for dehydration of ethanol. J. Membr. Sci..

[83] Zhu W.-P., Sun S.-P., Gao J., Fu F.-J., Chung T.-S. (2014). Dual-layer polybenzimidazole/polyethersulfone (PBI/PES) nanofiltration (NF) hollow fiber membranes for heavy metals removal from wastewater. J. Membr. Sci..

[84] Zhao L., Wu C., Liu Z., Zhang Q., Lu X. (2016). Highly porous PVDF hollow fiber membranes for VMD application by applying a simultaneous co-extrusion spinning process. J. Membr. Sci..

[85] Tsai H.-A., Chen Y.-L., Huang S.-H., Hu C.-C., Hung W.-S., Lee K.-R., Lai J.-Y. (2018). Preparation of polyamide/polyacrylonitrile composite hollow fiber membrane by synchronous procedure of spinning and interfacial polymerization. J. Membr. Sci..

[86] Ng D.Y.F., Wu B., Chen Y., Dong Z., Wang R. (2019). A novel thin film composite hollow fiber osmotic membrane with one-step prepared dual-layer substrate for sludge thickening. J. Membr. Sci..

[87] Naderi A., Chung T.-S., Weber M., Maletzko C. (2019). High performance dual-layer hollow fiber membrane of sulfonated pol-yphenylsulfone/Polybenzimidazole for hydrogen purification. J. Membr. Sci..

[88] Setiawan L., Shi L., Wang R. (2013). Dual layer composite nanofiltration hollow fiber membranes for low-pressure water softening. Polymer.

[89] Setiawan L., Wang R., Tan S., Shi L., Fane A.G. (2012). Fabrication of poly(amide-imide)-polyethersulfone dual layer hollow fiber membranes applied in forward osmosis by combined polyelectrolyte cross-linking and depositions. Desalination.

[90] Feng X., Jiang L.Y., Matsuura T., Wu P. (2016). Fabrication of hydrophobic/hydrophilic composite hollow fibers for DCMD: Influence of dope formulation and external coagulant. Desalination.

[91] Chwojnowski A., Wojciechowski C., Dudzin K., Lukowska E., Granicka L. (2009). New type of hollow fiber membrane for cell and microorganisms cultivation and encapsulation. Desalination.

[92] Çulfaz P.Z., Rolevink E., van Rijn C., Lammertink R.G., Wessling M. (2010). Microstructured hollow fibers for ultrafiltration. J. Membr. Sci..

[93] Çulfaz P., Wessling M., Lammertink R. (2010). Hollow fiber ultrafiltration membranes with microstructured inner skin. J. Membr. Sci..

